# High-fat diet induced obesity and anti-activin receptor antibody: Effects on bone properties in mice

**DOI:** 10.1016/j.bonr.2025.101878

**Published:** 2025-09-19

**Authors:** Frederik Duch Bromer, Andreas Lodberg, Lykke Sylow, Michala Carlsson, Christian Brix Folsted Andersen, Jesper Skovhus Thomsen, Annemarie Brüel

**Affiliations:** a Department of Biomedicine, Aarhus University, Aarhus, Denmark; b Department of Endocrinology and Internal Medicine, Aarhus University Hospital, Aarhus, Denmark; c Department of Clinical Medicine, Aarhus University, Aarhus, Denmark; d Department of Biomedical Sciences, Faculty of Health and Medical Sciences, University of Copenhagen, Copenhagen, Denmark

**Keywords:** Smad2/3, TGF-beta, Bone metabolism, Myostatin, Weight-loss

## Abstract

**Aim:**

Weight-loss therapy often results in an unintended loss of muscle and bone mass. Inhibitors of the activin receptor signaling pathway, such as bimagrumab, an anti-activin receptor antibody (αActRIIA/IIB ab), are under investigation to counteract weight-loss induced muscle loss, but their skeletal effects in obesity remain unclear. This study investigates αActRIIA/IIB ab on bone in mice exposed to a high-fat diet (HFD) model of obesity or standard chow.

**Materials and methods:**

Male C57BL/6 J mice were stratified into four groups (*n* = 10/group, standard chow or HFD for 10 weeks ± αActRIIA/IIB ab). αActRIIA/IIB ab (10 mg/kg) was administered twice weekly during the final three weeks. The femur and vertebral body were assessed using DEXA, μCT, mechanical testing, and histomorphometry.

**Results:**

HFD did not affect bone density, microstructure, or strength but reduced histological bone formation markers. In standard chow mice, αActRIIA/IIB ab increased trabecular bone volume fraction (BV/TV) and volumetric bone mineral density (vBMD) by 36 %. In HFD mice, the effect of αActRIIA/IIB ab was less pronounced but still increased BV/TV (+16 %) and vBMD (+13 %). For cortical bone, μCT parameters remained largely unaffected by αActRIIA/IIB ab, while the treatment increased periosteal mineralizing bone surfaces in standard chow mice (+217 %), but not in HFD mice.

**Conclusions:**

αActRIIA/IIB ab enhanced trabecular bone properties in standard chow-fed mice, but its anabolic effects were blunted in HFD-fed mice. Furthermore, αActRIIA/IIB ab improved cortical histological bone formation markers, while morphology remained unaffected, suggesting a site- or time-specific difference. Thus, αActRIIA/IIB ab holds potential for mitigating weight-loss-associated bone deterioration.

## Highlights

• Weight-loss therapies often cause an unintended loss of muscle and bone mass.
• Bimagrumab (αActRIIA/IIB ab) is being explored to preserve lean mass during weight loss, but its effects on bone are unclear.
• We investigated the effect of αActRIIA/IIB ab on bone in mice with obesity.
• αActRIIA/IIB ab improved trabecular bone structure, with minimal cortical bone changes.
• High-fat diet blunted bone anabolic effects, suggesting obesity may impair the bone response to activin receptor blockade.

## 1 Introduction

Obesity is defined by a high Body Mass Index and is a growing problem globally (Peeters et al., 2003; Abbafati et al., 2020). According to the World Health Organization, 18 % of adults worldwide are classified as obese, while 43 % are classified as overweight. Furthermore, the incidence of obesity is rising in both adults and children (Blüher, 2019). Obesity drastically reduces life expectancy due to its detrimental effects on multiple organ systems (Peeters et al., 2003). Over the years, the effect of obesity on bone properties has been debated. Increased mechanical load, as seen in obesity, is usually related to a higher bone mineral density (BMD) (Reid, 2002; Gkastaris et al., 2020). However, individuals suffering from obesity have a disproportionally higher fracture risk at certain skeletal sites (Fassio et al., 2018; Premaor et al., 2010).

Recent advancements in pharmaceuticals for treating obesity, such as glucagon like peptide-1 (GLP-1) receptor agonists, have attracted massive interest due to the ubiquitous nature of the obesity epidemic (Jastreboff and Kushner, 2023; Knudsen and Lau, 2019; Torekov et al., 2011). However, while these new drugs are effective in reducing total body mass, they often lead to an unwanted concurrent reduction in lean body mass (Wilding et al., 2021; Christoffersen et al., 2022). Moreover, treatment with GLP-1 receptor agonists has been shown to decrease BMD (Jensen et al., 2024). To mitigate this negative impact on skeletal muscle and bone integrity, combining GLP-1 receptor agonists with inhibitors of the activin-receptor signaling pathway (IASPs) is being explored.

IASPs are a new class of pharmaceuticals that have been shown to simultaneously increase skeletal muscle mass and improve bone density, microstructure, and strength (Pearsall et al., 2008; Lodberg, 2021; Brent et al., 2021; Lodberg et al., 2019; Bromer et al., 2025). As a group, they work by inhibiting activation of the activin receptor signaling pathways. The activin receptor is a multiunit complex that can be stimulated by different ligands e.g. activins, myostatin, and growth-differentiation factor-11 (GDF-11) (Lodberg, 2021; Sugino et al., 1988; Attisano et al., 1996; Whittemore et al., 2003; Rebbapragada et al., 2003). After ligand binding, the activin receptor-complex heteromerizes, and signaling is propagated through phosphorylation of the transcription factors Smad2 and Smad3, leading to the catabolic effect on muscle and bone tissue.

Bimagrumab is one such IASP – a human anti-activin receptor type IIA and type IIB antibody (αActRIIA/IIB ab) – which binds and blocks the ligand-binding site on the activin type IIA and type IIB receptors (Lach-Trifilieff et al., 2014). Originally developed to treat muscle atrophy (Amato et al., 2014), it is now being investigated in two phase 2 clinical trials in individuals with overweight and obesity: One in combination with the GLP-1 receptor agonist semaglutide (NCT05616013), and the other in combination with tirzepatide (NCT06643728), a combined GLP-1 receptor agonist and gastric inhibitory polypeptide (GIP) analogue. These clinical trials aim to evaluate whether bimagrumab can preserve lean body mass during weight reduction induced by semaglutide or tirzepatide (Nunn et al., 2024).

Multiple studies have shown the potency of αActRIIA/IIB ab to improve skeletal muscle mass in different diseases (Lach-Trifilieff et al., 2014; Morvan et al., 2017; Polkey et al., 2019; Hatakeyama et al., 2016; Rooks et al., 2020; Rooks et al., 2017a; Rooks et al., 2017b; Heymsfield et al., 2021). Recently, we demonstrated that αActRIIA/IIB ab can also improve bone parameters in both skeletally healthy and osteopenic female mice (Bromer et al., 2025). A newly registered phase 2 clinical trial (NCT05933499) aims to investigate the effect of bimagrumab on bone tissue in adults with obesity. However, despite the growing interest in αActRIIA/IIB ab for the treatment of obesity, no published data currently exist on the relationship between obesity-affected bone tissue and IASPs such as αActRIIA/IIB ab.

A commonly used preclinical animal model for studying obesity involves exposing mice to a high-fat diet (HFD). In this model, the mice rapidly gain total body weight (BW) and develop many obesity-associated complications, such as increased fat mass and decreased glucose tolerance (Nilsson et al., 2012; Fang et al., 2023; Nagy and Einwallner, 2018). In addition, a HFD has detrimental effects on bone, particularly on trabecular bone, in mice (Zhang et al., 2022), making it a suitable model for investigating whether αActRIIA/IIB ab can counteract obesity-induced skeletal deterioration.

Consequently, this study aimed to investigate the effects of anti-activin receptor antibody treatment on bone parameters in mice fed either a high-fat diet or a standard chow diet.

## 2 Materials and methods

The bones analyzed in the current study originate from a previously published study (Carlsson et al., 2025). The BW data were previously reported in that study, while all other data are original to the current publication.

### 2.1 Production of αActRIIA/IIB ab

Expression and purification were performed as described previously (Bromer et al., 2025; Carlsson et al., 2025). Briefly, a light chain and heavy chain of the αActRIIA/IIB ab were generated and transfected into Chinese hamster ovary (CHO) cells. The αActRIIA/IIB ab was then purified using affinity chromatography and exchanged into phosphate-buffered saline (PBS). Finally, its ability to inhibit the activity of activin A, myostatin, and GDF-11 was verified in a cell-based Smad-binding element luciferase assay, with inhibitory concentrations of 519 pM, 49 pM, and 143 pM, respectively.

### 2.2 Housing and animal procedures

After acclimatization, forty 14-week-old male C57BL/6 J mice (Janvier Labs, Le Genest-Saint-Isle, France) were allocated into four groups based on BW and body composition (*n* = 10 per group): Standard chow + vehicle (Chow), standard chow + αActRIIA/IIB ab (Chow + αActRIIA/IIB ab), high-fat diet + vehicle (HFD), high-fat diet + αActRIIA/IIB ab (HFD + αActRIIA/IIB ab). The mice had ad libitum access to either a standard chow diet (Altromin no. 1324; Brogaarden, Hørsholm, Denmark) containing 11 kcal% fat and plain drinking water without added sucrose or a 45 kcal% high-fat diet (Research diet no. D12451 2.5HS; Brogaarden, Hørsholm, Denmark) and 10 % sucrose water throughout the study. Additional dietary content can be found in supplementary information S1. All animals were housed in individual cages with a 12 h day/night cycle and a thermoneutral temperature of 30 °C.

Seven weeks after the start of their respective diets, animals in the αActRIIA/IIB ab groups received intraperitoneal injections of αActRIIA/IIB ab (10 mg/kg) twice a week for three weeks. Animals in the vehicle groups received intraperitoneal injections of equal volume vehicle (PBS) twice a week. To monitor the effect of both diet and treatment, BW was measured weekly. To facilitate dynamic bone histomorphometry, tetracycline (20 mg/kg; T3383, Sigma-Aldrich, St. Louis, MO, USA) and alizarin red (20 mg/kg; A3882; Sigma-Aldrich, St. Louis, MO, USA) was injected subcutaneously (s.c.) 8 and 4 days before euthanasia, respectively.

After 3 weeks of treatment, all animals were sedated with a pentobarbital/lidocaine mix and euthanized by cervical dislocation. The right femora were removed, their length was measured using a digital caliper, and they were then frozen in Ringer's solution at −20 °C. The left femora were removed, fixed in 4 % formaldehyde for 48 h, and then transferred to 70 % ethanol. The L5 and L6 vertebra were removed and frozen in Ringer's solution at −20 °C.

**Fig. 1:**
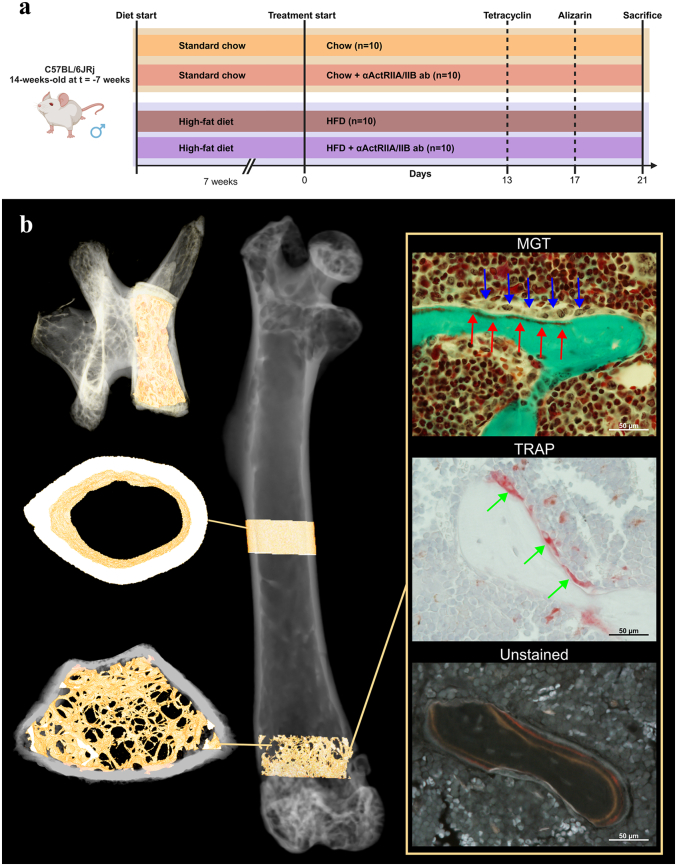
Study overview. (a) Timeline of the study. (b) 3D semitransparent renderings of a femur and a vertebra highlighting the volumes of interests (VOIs) in the femoral mid-diaphysis, distal femoral metaphysis, and vertebral body. In addition, histological sections from the distal femoral metaphysis are shown. The first section is stained with Masson-Goldner trichrome (MGT), where blue arrows indicate osteoblasts and red arrows indicate osteoid. The second section is stained for tartrate resistant acid phosphatase (TRAP), with green arrows pointing to multinucleated osteoclasts. Lastly, an unstained section viewed under fluorescence microscopy showing a red alizarin label and an orange tetracycline label.

A timeline of the experiment is shown in Fig. 1a. The study was approved by the Danish Animal Experiments Inspectorate (2021-15-0201-01085). No animals died prematurely.

### 2.3 Dual-energy X-ray absorptiometry (DEXA)

Areal bone mineral density (aBMD) and bone mineral content (BMC) was determined for the whole femur using DEXA (Sabre XL; Nordland Stratec, Pfortzheim, Germany). Scans were performed at a spatial resolution of 0.1 × 0.1 mm^2^ with a scan speed of 3 mm/s. Daily quality assurance was performed using the phantoms supplied with the scanner.

### 2.4 Micro-computed tomography

Bone microstructural parameters were assessed using micro-computed tomography (μCT) (μCT35; Scanco Medical Ag, Brüttisellen, Switzerland) for the right distal femoral metaphyses, the L5 vertebrae, and the right femoral mid-diaphyses. The distal femoral metaphyses and the L5 vertebrae were imaged with 1000 projections / 180° using an X-ray potential of 55 kVp, an isotropic voxel size of 3.5 μm, and an integration time of 800 ms. For the mid-diaphyses, imaging was performed with 500 projections / 180° using an X-ray potential of 55 kVp, an isotropic voxel size of 7 μm, and an integration time of 300 ms. Software supplied with the scanner was used to demarcate the volumes of interest (VOI) for each bone site (Fig. 1b). For the distal femoral metaphyses, a 1000-μm-high VOI encompassing only the trabecular region and excluding the cortex was placed 200 μm proximal to the most proximal part of the growth plate. For the L5 vertebrae, the VOI included the entire vertebral body, excluding cortical bone. For the femoral mid-diaphyses, an 820-μm-high VOI was centered on the midpoint of the diaphysis, including the entire bone and marrow volume.

After reconstruction, a low-pass Gaussian filter (σ = 0.8, support = 1) was applied to the data followed by segmentation using a threshold of 531 mg hydroxyapatite (HA)/cm^3^ for the distal femoral metaphysis and the L5 vertebra, and 595 mg HA/cm^3^ for the mid-diaphysis. At trabecular sites, bone volume fraction (BV/TV), trabecular thickness (Tb.Th), trabecular number (Tb.N), trabecular spacing (Tb.Sp), structure model index (SMI), volumetric bone mineral density (vBMD), and tissue mineral density (TMD) were determined. At the femoral mid-diaphysis, bone area (B·Ar), tissue area (T.Ar), marrow area (M.Ar), cortical thickness (Ct.Th), and TMD were determined.

### 2.5 Mechanical testing

Bone strength (maximum applied force, *F*_max_) was determined by applying downwards force at a constant deflection rate of 2 mm/min to the bone until fracture using a materials testing machine (5566; Instron, High Wycombe, UK). For the femoral mid-diaphyses, a three-point-bending test was performed by placing the bone on two parallel rounded rods with a distance of 7 mm, while a third rounded rod applied downwards force at the midpoint between the two supporting rods until bone fracture. Subsequently, the proximal part of the femur was fixed in a custom-made testing jig, and downwards force was applied to the femoral head at a constant rate of 2 mm/min until fracture of the femoral neck. The L5 vertebral bodies were prepared by removing all bone processes and both endplates. Afterwards, the vertebral bodies were placed in the materials testing machine and downwards force was applied at a constant rate of 2 mm/min until fracture. The load-deformation curves were analyzed using in-house-developed software.

### 2.6 Tissue preparation for histological analysis

The left distal femora and the L6 vertebra were dehydrated and embedded undecalcified in methylmetacrylate, cut into 7-μm-thick longitudinal sections, and mounted on glass slides. The femoral samples were then either left unstained for dynamic bone histomorphometry, stained with Masson-Goldner trichrome, or enzymatically stained for tartrate-resistant acid phosphatase (TRAP), while the L6 vertebra samples were either left unstained or stained with Masson-Goldner trichrome. After mechanical testing of the femur, a 200-μm-thick cross section was cut from the femoral mid-diaphysis, mounted on glass slides, and left unstained for dynamic histomorphometry.

### 2.7 Dynamic bone histomorphometry

The trabecular bone of the distal femoral metaphysis was analyzed using a 1000-μm-high region of interest (ROI) located 200 μm proximal to the most proximal part of the distal growth plate. The ROI of the L6 vertebra comprised the entire trabecular region of the vertebral body, excluding the primary spongiosa. Using a fluorescent microscope (Eclipse i80; Nikon, Tokyo, Japan) connected to a computer with stereology software (Visiopharm v. 2020.09.0.8195; Visiopharm, Hørsholm, Denmark), a randomly oriented line grid was superimposed digitally over the samples, and intersections between the lines and bone or fluorescent labels were counted by a blinded observer. Cortical bone was analyzed using the cross-sectional samples of the femoral mid-diaphysis, where a grid with 24 lines radiating from the center was superimposed over the center of the mid-diaphysis. The intersections between the lines and bone or labels were then counted.

For both trabecular bone and cortical bone, mineralizing bone surfaces (MS/BS), mineral apposition rate (MAR), and bone formation rate (BFR/MS) were determined (Dempster et al., 2013). For cortical samples without visible double labels, a MAR and BFR/BS value of 0 was imputed, since all animals had double labels in trabecular bone, indicating that no injections were missed.

### 2.8 Osteoblast, osteoid, and osteoclast count

For femoral samples, osteoblast-covered bone surfaces (Ob.S/BS) and osteoid-covered bone surfaces (OS/BS) were determined using the Masson-Goldner trichrome stained sections, while osteoclast-covered bone surfaces (Oc.S/BS) were determined using the sections stained for TRAP. For vertebral samples, only OS/BS was estimated, as the samples had not been fixed in 4 % formaldehyde after removal. ROIs were delineated, as described above. Osteoblasts were identified as clear, cuboidal cells on the bone surface, osteoid as a red seam just beneath the bone surface, and osteoclasts as multinucleated, TRAP-positive cells on the bone surface.

### 2.9 Statistical analysis

Statistical analysis was performed using GraphPad Prism 10.0.1. Normality of the data was assessed using QQ-plots of the residuals. For normally distributed data, a two-way analysis of variance (2 W-ANOVA) was performed, followed by a Holm-Sidak analysis for multiple comparison for Chow vs. HFD, Chow + αActRIIA/IIB ab vs. Chow, and HFD + αActRIIA/IIB ab vs. HFD. For non-normally distributed data, a Kruskal-Wallis test was performed followed by a Dunn's test. Data are presented as relative values in the text and as mean ± standard deviation (SD) in the figures and tables. In the analyses, *p* < 0.05 was considered statistically significant.

## 3 Results

### 3.1 Body weight and femoral length

**Fig. 2:**
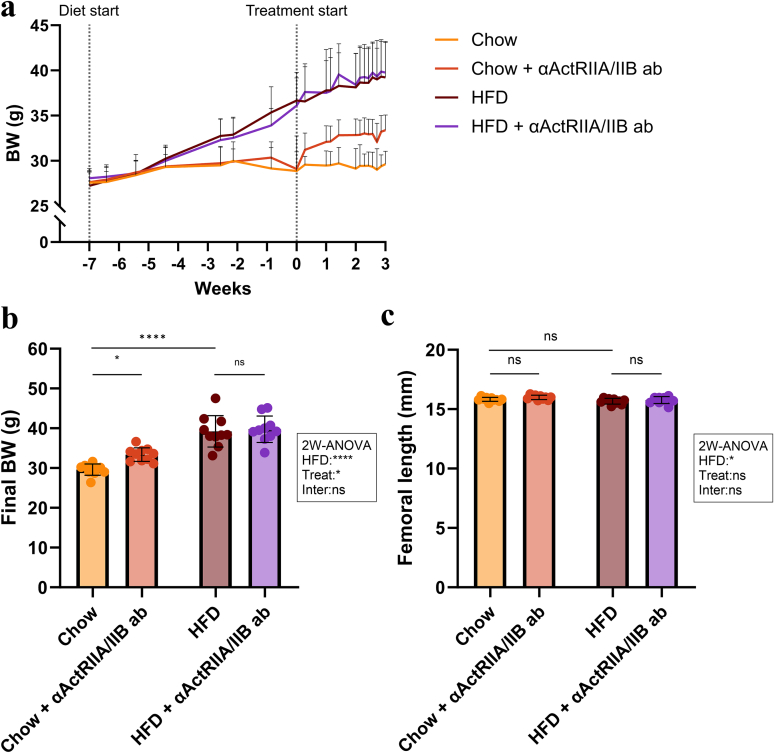
Body weight and femoral length. (a) Body weight (BW) during the study. (b) Final BW. (c) Femoral length. Data is presented as mean ± SD. ^⁎^: *p* < 0.05. ^⁎⁎⁎⁎^: *p* < 0.0001. ns: Not significant. Group effects of high-fat diet (HFD), αActRIIA/IIB ab treatment (Treat), and interaction between the two (Inter) were determined by two-way analysis of variance (2 W-ANOVA) and are shown in the textbox. Chow diet and HFD contained 11 and 45 kcal% fat, respectively.

The final BW of HFD mice was higher (+32 %) than that of Chow mice (Fig. 2a-b). αActRIIA/IIB ab increased final BW in Chow mice (+13 %), but did not affect BW in HFD mice.

The femoral length did not differ between the groups, regardless of diet or treatment (Fig. 2c).

### 3.2 Dual-energy X-ray absorptiometry

Whole femur BMC and aBMD did not differ between the separate groups regardless of diet or treatment, however, the 2 W-ANOVA detected a significant increase in BMC due to treatment (Table 1).

**Table 1:** Bone properties. Areal bone mineral density (aBMD) and bone mineral content (BMC) determined by Dual-Energy X-Ray Absorptiometry (DEXA). Trabecular separation (Tb.Sp), tissue mineral density (vBMD), cortical bone area (Ct.B.Ar), cortical tissue area (Ct.T.Ar), cortical marrow area (Ct.Ma.Ar), and cortical thickness (Ct.Th) determined by micro-computed tomography (μCT). Bone strength (*F*_max_) at the femoral neck, L5 vertebral body, and femoral mid-diaphysis. ns: Not significant. Group effect of high-fat diet (HFD), treatment (Treat), and interaction between the two (Inter) from the two-way analysis of variance (2 W-ANOVA) is shown in the textbox. Data is presented as mean (SD). ^a^: *p* < 0.05 Chow vs. HFD. ^b^: *p* < 0.05 Chow vs. Chow + αActRIIA/IIB ab. ^c^: *p* < 0.05 HFD vs. HFD + αActRIIA/IIB ab. Group effects of high-fat diet (HFD), αActRIIA/IIB ab treatment (Treat), and interaction between the two (Inter) were determined by two-way analysis of variance (2 W-ANOVA) and are shown for each parameter. *: *p* < 0.05, **: *p* < 0.01, ***: *p* < 0.001, ****: *p* < 0.0001, ns: not significant.

	Chow	Chow + αActRIIA/IIB ab	HFD	HFD + αActRIIA/IIB ab	2 W-ANOVA		
					HFD	Treat	Inter
Whole femur							
aBMD (mg/cm^3^)	61 (2)	64 (3)	63 (3)	63 (3)	ns	ns	ns
BMC (mg/cm^3^)	24 (1)	26 (2)	24 (2)	25 (1)	ns	*	ns

Distal femoral metaphysis							
Tb.Sp (μm)	213 (12)	187 (9)b	201 (25)	191 (9)	ns	***	ns
TMD (mg/cm3)	902 (8)	920 (6)b	946 (10)a	947 (12)	****	**	**

L5 vertebral body							
Tb.Sp (μm)	173 (7)	161 (9)b	163 (13)	157 (8)	*	**	ns
TMD (mg/cm3)	906 (7)	915 (8)b	942 (9)a	944 (6)	****	*	ns

Femoral mid-diaphysis							
Ct.Ba.Ar (mm2)	0.97 (0.03)	0.97 (0.05)	0.96 (0.05)	0.99 (0.04)	ns	ns	ns
Ct.T.Ar (mm2)	2.16 (0.12)	2.10 (0.13)	2.08 (0.07)	2.17 (0.11)	ns	ns	*
Ct.Ma.Ar (mm2)	1.19 (0.12)	1.13 (0.10)	1.13 (0.07)	1.18 (0.08)	ns	ns	ns
Ct.Th (μm)	201 (9)	206 (9)	203 (12)	207 (8)	ns	ns	ns
TMD (mg/cm3)	1153 (8)	1152 (9)	1163 (12)	1160 (11)	**	ns	ns

Mechanical strength							
Femoral neck, *F*_max_ (N)	12.9 (1.7)	13.9 (1.3)	13.6 (1.2)	14.3 (1.7)	ns	ns	ns
Femoral neck, stiffness (N/mm)	135.6 (21.7)	152.9 (20.3)	142.0 (29.7)	153.7 (24.4)	ns	ns	ns
L5 compression, *F*_max_ (N)	20.9 (2.7)	24.4 (4.3)	23.6 (5.4)	23.7 (4.7)	ns	ns	ns
L5 compression, stiffness (N/mm)	184.8 (54.7)	226.7 (57.0)	239.2 (83.8)	254.0 (101.8)	ns	ns	ns
Mid-diaphysis, *F*_max_ (N)	17.6 (1.8)	17.9 (1.5)	18.1 (1.5)	18.1 (1.7)	ns	ns	ns
Mid-diaphysis, stiffness (N/mm)	91.6 (8.6)	93.1 (6.3)	95.4 (8.8)	97.9 (10.3)	ns	ns	ns

### 3.3 Micro-computed tomography

#### 3.3.1 Distal femoral metaphysis

**Fig. 3:**
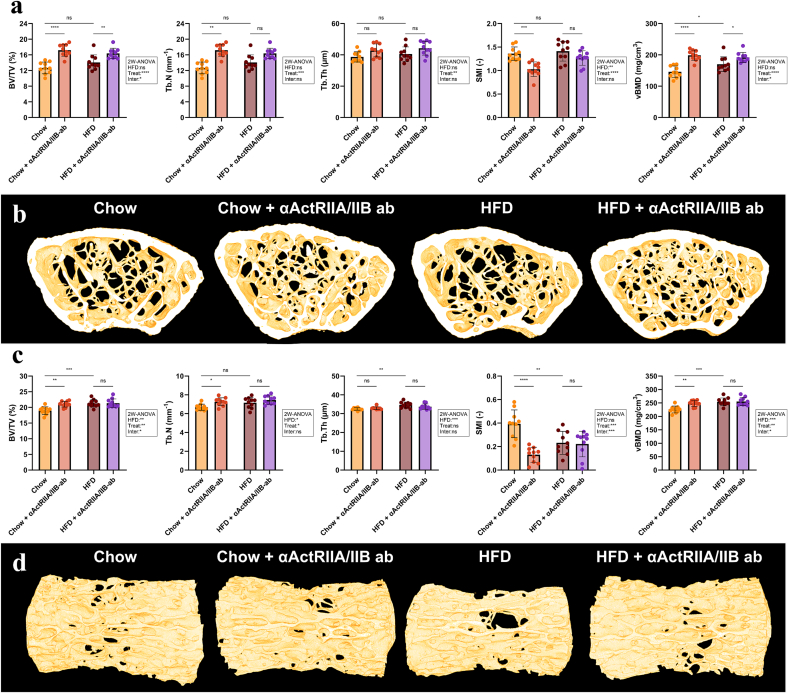
Trabecular μCT parameters. (a) μCT bone parameters at the distal femoral metaphysis. (b) Representative μCT reconstructions of the distal femoral metaphysis. (c) μCT bone parameters at the L5 vertebral body. (d) Representative μCT reconstructions of the L5 vertebral body. Bone volume fraction (BV/TV), trabecular number (Tb.N), trabecular thickness (Tb.Th), structure model index (SMI), and volumetric bone mineral density (vBMD). Data is presented as mean ± SD. ^⁎^: *p* < 0.05. ^⁎⁎^: *p* < 0.01. ^⁎⁎⁎^: *p* < 0.001. ^⁎⁎⁎⁎^: *p* < 0.0001. ns: Not significant. Group effects of high-fat diet (HFD), αActRIIA/IIB ab treatment (Treat), and interaction between the two (Inter) were determined by two-way analysis of variance (2 W-ANOVA) and are shown in textbox. Chow diet and HFD contained 11 and 45 kcal% fat, respectively.

HFD mice had increased vBMD (+16 %) and TMD (+5 %) compared to Chow mice, but no other trabecular parameters were affected by the diet (Fig. 3a-b, Table 1). αActRIIA/IIB ab treatment improved trabecular bone structure in Chow mice, as demonstrated by an increase in BV/TV (+36 %), Tb.N (+14 %), vBMD (+36 %), and TMD (+2 %). Furthermore, αActRIIA/IIB ab treatment decreased Tb.Sp (−12 %) and SMI (−24 %), indicating a beneficial shift in the architecture of the trabecular network towards a more plate-like structure. In HFD mice, αActRIIA/IIB ab treatment modestly improved trabecular bone structure, increasing BV/TV (+16 %) and vBMD (+13 %). However, unlike in chow-fed mice, it did not significantly alter Tb.N, Tb.Th, Tb.Sp, SMI, or TMD. Statistically significant interactions were observed between diet and treatment for BV/TV, vBMD, and TMD, indicating that HFD negatively affected the bone anabolic effect of αActRIIA/IIB ab treatment.

#### 3.3.2 L5 vertebra

HFD increased BV/TV (+12 %), Tb.Th (+7 %), vBMD (+13 %), and TMD (+4 %) compared to Chow, while SMI (−41 %) was decreased in HFD compared to Chow (Fig. 3c-d, Table 1). In Chow mice, αActRIIA/IIB ab treatment increased BV/TV (+11 %), Tb.N (+9 %), vBMD (+10 %), and TMD (+1 %), and decreased Tb.Sp (−7 %) and SMI (−67 %), whereas αActRIIA/IIB ab treatment did not affect any microstructural parameter in HFD mice.

Similarly to trabecular bone at the metaphysis, a statistically significant interaction was observed between diet and treatment for BV/TV, vBMD, and SMI, with HFD blunting the anabolic effect of αActRIIA/IIB ab treatment.

#### 3.3.3 Femoral mid-diaphysis

Neither diet nor αActRIIA/IIB ab treatment altered B·Ar, T.Ar, M.Ar, or Ct.Th at the femoral mid-diaphysis (Table 1), indicating that the cortical microstructure was unaffected by both diet and 3 weeks of αActRIIA/IIB ab treatment.

Taken together, the μCT-derived findings show that HFD slightly elevated trabecular bone parameters, while αActRIIA/IIB ab treatment improved trabecular bone structure in both Chow and HFD mice, with a less pronounced effect in HFD mice. Moreover, neither HFD nor αActRIIA/IIB ab treatment significantly affected the cortical bone morphology.

### 3.4 Mechanical testing

Neither diet nor αActRIIA/IIB ab treatment had a statistically significant effect on bone strength at the femoral mid-diaphysis, femoral neck, or L5 vertebral body (Table 1).

### 3.5 Dynamic bone histomorphometry

#### 3.5.1 Distal femoral metaphysis

While HFD did not significantly affect either MS/BS, MAR, or BFR/BS compared to Chow, a borderline significant reduction in MS/BS (−47 %, *p* = 0.07) was observed in HFD compared to Chow (Table S1). Although αActRIIA/IIB ab did not result in significant difference in MS/BS, MAR, or BFR/BS between separate groups, the 2 W-ANOVA did detect a significant increase in MS/BS and BFR/BS due to the treatment.

#### 3.5.2 L6 vertebra

HFD reduced MS/BS (−44 %) and BFR/BS (−46 %) but did not affect MAR compared to Chow mice (Table S1). αActRIIA/IIB ab treatment did not affect MS/BS, MAR, or BFR/BS in Chow mice, but increased both MS/BS (+35 %) and BFR/BS (+39 %) in HFD mice.

#### 3.5.3 Femoral mid-diaphysis

**Fig. 4:**
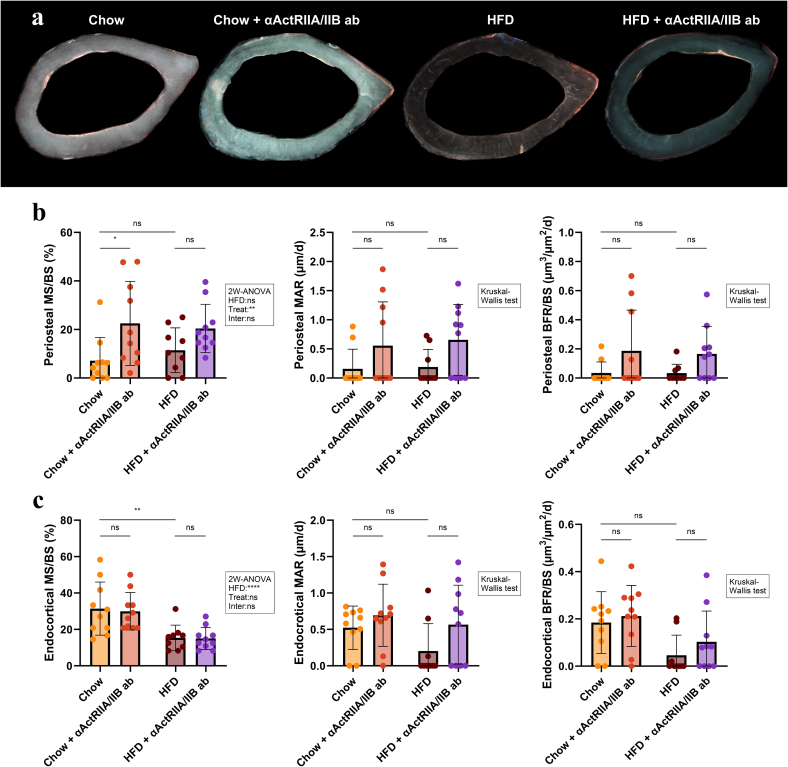
Dynamic bone histomorphometry at the femoral mid-diaphysis. (a) A representative sample from each group. Yellow is the tetracycline label and red is the alizarin label. Mineralizing bone surfaces (MS/BS), mineral apposition rate (MAR), and bone formation rate (BFR/BS) for the (b) periosteal and (c) endosteal mid-diaphyseal bone surface. Data is presented as mean ± SD. *: p < 0.05, **: p < 0.01, ****: p < 0.0001, ns: Not significant. Group effects of high-fat diet (HFD), αActRIIA/IIB ab treatment (Treat), and interaction between the two (Inter) were determined by two-way analysis of variance (2 W-ANOVA) and are shown in the textbox. Chow diet and HFD contained 11 and 45 kcal% fat, respectively.

At the periosteal bone surface, MS/BS, MAR, and BFR/BS did not differ between HFD and Chow mice (Fig. 4a,b). Chow mice treated with αActRIIA/IIB ab had higher MS/BS (+217 %) than their vehicle treated controls, while MAR and BFR/BS remained unaffected. Conversely, in HFD mice, the treatment did not significantly affect the periosteal bone surface. At the endocortical bone surface, HFD decreased MS/BS (−51 %) but did not affect MAR or BFR/BS compared to Chow mice (Fig. 4a,c). Treatment with αActRIIA/IIB ab had no effect on bone formation markers at the endocortical bone surface in either HFD or Chow mice.

These dynamic bone histomorphometry findings illustrate that HFD decreased trabecular MS/BS at the L6 vertebral body and at the endocortical envelope of the femoral mid-diaphysis. In contrast, treatment with αActRIIA/IIB ab increased MS/BS for trabecular bone and at the periosteal cortical bone surface but had no effect on the endosteal cortical bone surface.

### 3.6 Osteoblast, osteoid, and osteoclast count

#### 3.6.1 Distal femoral metaphysis

Both Ob.S/BS and OS/BS were significantly lower (−79 % and − 73 %, respectively) in HFD mice compared to Chow mice (Table S1). In Chow mice, αActRIIA/IIB ab treatment significantly decreased Ob.S/BS (−66 %), whereas no difference in Ob.S/BS was found in HFD mice. Additionally, treatment with αActRIIA/IIB ab did not affect OS/BS in either diet group. Furthermore, Oc.S/BS was unaffected by both diet and αActRIIA/IIB ab treatment.

#### 3.6.2 L6 vertebra

OS/BS was lower (−54 %) in HFD compared to Chow mice (Table S1). In Chow animals, αActRIIA/IIB ab treatment significantly reduced OS/BS (31 %), whereas the treatment had no effect on OS/BS in HFD animals. Ob.S/BS and Oc.S/BS could not be measured, as described in M&M 2.8.

## 4 Discussion

In this study, we investigated the effects of anti-activin receptor antibody treatment on bone parameters in mice exposed to either a high-fat diet or a standard chow diet. We found that treatment with αActRIIA/IIB ab in mice fed a standard chow resulted in significantly improved trabecular bone microstructure at both the distal femoral metaphysis and the L5 vertebral body. Interestingly, the anabolic effect of αActRIIA/IIB ab treatment was less pronounced in mice exposed to a high-fat diet, with significant negative interactions between treatment and high-fat diet for several parameters, including metaphyseal BV/TV and vBMD and vertebral BV/TV, SMI, and vBMD. This suggests that the high-fat diet blunted the bone anabolic effect of αActRIIA/IIB ab. The trabecular bone formation in this study appears to be driven mainly by αActRIIA/IIB ab osteoblastic stimulation, whereas no effect was observed on osteoclast-covered surfaces. Although activin A stimulation of osteoclast differentiation and activity has been shown *in vitro* (Fuller et al., 2000; Sakai et al., 1993) and some studies of IASPs have found suppression of osteoclast-covered bone surfaces (Brent et al., 2021; Jeong et al., 2018; Lotinun et al., 2010), multiple other *in vivo* studies have found unaffected osteoclast-covered surfaces (Bromer et al., 2025; Lodberg et al., 2018; Chantry et al., 2010).

The high-fat diet model used in the current study did not negatively affect bone density, microstructural properties, or strength. However, the high-fat diet resulted in reduced histological bone formation markers at multiple bone sites, including the femoral metaphysis, vertebral body, and the mid-diaphyseal endocortical bone, as well as decreased osteoblast- and osteoid-covered surfaces. These findings suggest that an extended study duration could result in significantly impaired structural bone properties. Counterintuitively, high-fat diet increased trabecular bone parameters at the L5 vertebra compared to controls, while dynamic histomorphometry revealed reduced bone formation. Increased body weight is known to enhance mechanical loading, which may explain the higher static trabecular bone values. A plausible explanation is that bone formation was initially stimulated by the increased mechanical load but declined with prolonged high-fat diet, ultimately leading to negative effects on bone, as captured by the dynamic bone histomorphometry performed at the study endpoint. This interpretation is consistent with the two-phase skeletal response to high-fat diet reported by Lecka-Czernik et al. (Lecka-Czernik et al., 2015), and with observations by Zhang et al. that longer high-fat diet exposure is associated with detrimental effects on bone (Zhang et al., 2022).

Unlike trabecular bone, cortical bone parameters were mostly unaffected by αActRIIA/IIB ab treatment, with only a slight increase in BMC detected by the two-way analysis of variance. This discrepancy between trabecular and cortical bone responses is consistent with our previous findings in female mice, where αActRIIA/IIB ab significantly improved trabecular microstructure but led to only modest increases in aBMD, BMC, and microstructural cortical parameters (B.Ar and T.Ar) in mice of similar age and strain (Bromer et al., 2025). The anabolic effect of αActRIIA/IIB ab on cortical bone is supported by the observed increase in histological bone formation markers at the periosteal, but not endocortical, bone surface, aligning with our previous findings (Bromer et al., 2025). Interestingly, this pattern contrasts with the cortical effects of the high-fat diet in the present study, which preferentially affected the endocortical surface without influencing periosteal bone. This site-specific difference in cortical response should be considered when evaluating the therapeutic potential of activin receptor inhibition in obesity-related bone disease, although it may reflect species-specific effects and warrants investigation in humans. The difference in trabecular and cortical bone response contrasts notably with studies showing that ligand trap IASPs, which target and bind the ligands instead of the receptor, substantially improve both cortical and trabecular bone parameters (Pearsall et al., 2008; Brent et al., 2021; Lodberg et al., 2018). Altogether, the difference in effect on cortical and trabecular bone may reflect the slower bone turnover at cortical bone sites or indicate a site-specific variation in the anabolic response to αActRIIA/IIB ab treatment.

Initially, IASPs were explored for their bone anabolic potential. However, after their hematopoietic and muscle anabolic effects were discovered, clinical research shifted focus towards diseases affecting muscle and blood tissue (Lodberg, 2021). With growing interest in IASPs for alleviating muscle loss induced by weight loss pharmaceutical, attention is now returning to their effects on bone tissue, particularly in bones affected by obesity. To our knowledge, the current study is the first to investigate the effects of IASPs on bone in an obesity model. The high-fat diet caused a substantial increase in body weight and fat mass and induced obesity-related diabetic changes, as previously reported by Michala et al. (Carlsson et al., 2025) However, the bone parameters analyzed in the current study using DEXA, μCT, and mechanical testing were largely unaffected by the high-fat diet. This was somewhat unexpected, as multiple studies have demonstrated that C57 mice are susceptible to bone deterioration from a high-fat diet, as highlighted in a systematic review by Zhang et al. (Zhang et al., 2022) Their analysis indicated that male mice, mice aged 6 to 12 weeks at study initiation, and studies with a duration of 10 weeks all showed a significant decrease in femoral metaphyseal bone volume fraction. These study parameters were mostly consistent with the design of the current study, although the mice in the present study were slightly older (14 weeks) than the optimal age-range (6–12 weeks) described by Zhang et al. As mice begin to exit their period of rapid skeletal growth around this age, this may explain the divergent effects on trabecular bone. However, Zhang et al. also found that diets containing more than 50 % of calories from fat were more effective at inducing losses in trabecular bone volume fraction. In contrast, our study used a diet with 45 kcal% fat content, which may partially explain the absence of a significant deteriorating effect. Finally, Zhang et al. found evidence that a longer duration of high-fat diet could further impair trabecular bone parameters, which aligns with the decreased bone formation markers from the dynamic bone histomorphometry analysis, as these markers are generally altered before the changes manifest in bone density and strength. Furthermore, there is evidence that the composition of the diet, rather than just the fat percentage, plays an important role in murine high-fat diets, which could also have influenced the findings (Beaver et al., 2023). However, despite the absence of bone-deteriorating effects from the high-fat diet in our study, we detected a statistically significant negative interaction between the diet and the bone anabolic effect of αActRIIA/IIB ab.

Contrary to expectation, Chow mice treated with αActRIIA/IIB ab had lower Ob.S/BS and OS/BS than their controls. In contrast, we previously found a slight increase in both Ob.S/BS and OS/BS in female mice treated with αActRIIA/IIB ab^18^. This difference in response to αActRIIA/IIB ab may be sex- or age-related, however, the other bone parameters in the current study showed otherwise comparable bone anabolic effects to those found in our earlier study, suggesting little to no sex-related difference. Current bone anabolic therapy for men is restricted to intermittent parathyroid hormone due to the increased incidence of cardiovascular events reported with anti-sclerostin antibody (Saag et al., 2017). αActRIIA/IIB may represent a viable alternative, particularly as sex hormone levels in men appear to be unaffected by treatment (Garito et al., 2018).

In conclusion, αActRIIA/IIB ab treatment increased cortical bone formation markers and caused a potent bone anabolic response in the trabecular bone of male mice exposed to a standard diet, but this response was blunted in mice exposed to a high-fat diet.

## CRediT authorship contribution statement

**Frederik Duch Bromer:** Conceptualization, Formal analysis, Investigation, Methodology, Visualization, Writing – original draft. **Andreas Lodberg:** Conceptualization, Methodology, Supervision, Writing – review & editing. **Lykke Sylow:** Conceptualization, Investigation, Writing – review & editing. **Michala Carlsson:** Conceptualization, Investigation, Writing – review & editing. **Christian Brix Folsted Andersen:** Resources, Writing – review & editing. **Jesper Skovhus Thomsen:** Conceptualization, Methodology, Supervision, Writing – review & editing. **Annemarie Brüel:** Conceptualization, Methodology, Supervision, Writing – review & editing.

## Declaration of competing interest

AL has served as a consultant or has received advisory fees from Acarios, Aureka Biotechnologies, Bluejay Therapeutics, Epirium Bio, and Morgan Stanley. AL, AM, and JST have performed sponsored research for Keros Therapeutics. LS owns stock in Eli Lilly and Company.

## Data Availability

Data will be made available on request.

## Glossary

2W-ANOVA: Two-way analysis of variance
ANOVA: Analysis of variance
B.Ar: Bone area
BFR/BS: Bone formation rate per bone surface
BMC: Bone mineral content
BMI: Body mass index
BS: Bone surface
Ct.Th: Cortical thickness
DEXA: Dual-energy X-ray absorptiometry
Fmax: Maximum applied force
GDF-11: Growth-differentiation factor-11
GLP-1: Glucagon-like peptide-1
HA: Hydroxyapatite
HFD: High-fat diet
IASP: Inhibitor of the activin receptor signalling pathway
M.Ar: Marrow area
MAR: Mineral apposition rate
MS/BS: Mineralising surface per bone surface
OS/BS: Osteoid-covered bone surface
Ob.S/BS: Osteoblast-covered bone surface
Oc.S/BS: Osteoclast-covered bone surface
PBS: Phosphate-buffered saline
ROI: Region of interest
SD: Standard deviation
SMI: Structure model index
T.Ar: Tissue area
TGF-beta: Transforming growth factor-beta
TMD: Tissue mineral density
TRAP: Tartrate-resistant acid phosphatase
Tb.N: Trabecular number
Tb.Sp: Trabecular spacing
Tb.Th: Trabecular thickness
VOI: Volume of interest
aBMD: Areal bone mineral density
vBMD: Volumetric bone mineral density
αActRIIA/IIB ab: Anti-activin receptor type IIA and type IIB antibody (bimagrumab)

## Appendix A Supplementary data

Supplementary material Image 1
