# Supplementary material for: High-fat diet induced obesity and anti-activin receptor antibody: Effects on bone properties in mice
Source: Bone Rep. 2025 Sep 19;27:101878. doi: 10.1016/j.bonr.2025.101878 (PMC12493230; doi:10.1016/j.bonr.2025.101878)
Supplement: Supplementary file 1 — Supplementary material [file mmc1.docx]

**Supplementary table 1** Histological properties at the distal femoral metaphysis and the L6 vertebral body. Mineralizing surfaces (MS/BS), mineral apposition rate (MAR), bone formation rate (BFR/BS), osteoblast-covered bone surfaces (Ob.S/BS), osteoid-covered bone surfaces (OS/BS), and osteoclast-covered bone surfaces (OC/BS). Data is presented as mean (SD). ^a^: *p* < 0.05 Chow vs. HFD. ^b^: *p* < 0.05 Chow vs. Chow + αActRIIA/IIB ab. ^c^: *p* < 0.05 HFD vs. HFD + αActRIIA/IIB ab. Group effects of high-fat diet (HFD), αActRIIA/IIB ab treatment (Treat), and interaction between the two (Inter) were determined by two-way analysis of variance (2W-ANOVA) and are shown for each parameter. *: *p* < 0.05, **: *p* < 0.01, ***: *p* < 0.001, ****: *p* < 0.0001, ns: not significant.

|  | **Chow** | Chow +  αActRIIA/IIB ab | **HFD** | HFD + αActRIIA/IIB ab | 2W-ANOVA | | | | | |
| --- | --- | --- | --- | --- | --- | --- | --- | --- | --- | --- |
|  |  |  |  |  | HFD | | Treat | | Inter | |
| ***Distal femoral metaphysis*** | | | | | | | | | |  |
| **MS/BS (%)** | **25 (7)** | **32 (13)** | **13 (5)** | **25 (14)** | ***** | | ****** | | **ns** | |
| **MAR (µm/d)** | **1.2 (0.1)** | **1.3 (0.2)** | **1.3 (0.3)** | **1.3 (0.3)** | **ns** | | **ns** | | **ns** | |
| **BFR/BS (µm^3^/µm^2^/d)** | **0.30 (0.09)** | **0.41 (0.21)** | **0.17 (0.07)** | **0.37 (0.28)** | **ns** | | ***** | | **ns** | |
| **Ob.S/BS (%)** | **11 (4)** | **4 (3)^b^** | **2 (1)^a^** | **5 (4)** | ****** | | ***** | | ******* | |
| **OS/BS (%)** | **12 (5)** | **6 (5)** | **3 (1)^a^** | **9 (9)** | **ns** | | **ns** | | ****** | |
| **OC/BS (%)** | **16 (5)** | **22 (5)** | **18 (6)** | **16 (6)** | **ns** | | **ns** | | **ns** | |
|  |  |  |  |  |  |  | |  | | |
| ***L6 vertebral body*** | | | | | | | | | |  |
| **MS/BS (%)** | **30 (2)** | **30 (4)** | **17 (6)^a^** | **23 (5)^c^** | ******** | | ***** | | **Ns** | |
| **MAR (µm/d)** | **1.2 (0.2)** | **1.1 (0.1)** | **1.2 (0.2)** | **1.2 (0.2)** | **ns** | | **ns** | | **ns** | |
| **BFR/BS (µm^3^/µm^2^/d)** | **0.35 (0.05)** | **0.34 (0.05)** | **0.19 (0.08)^a^** | **0.26 (0.06)^c^** | ******** | | **ns** | | ***** | |
| **OS/BS (%)** | **17 (4)** | **12 (3)^b^** | **8 (4)^a^** | **8 (2)** | ******** | | ***** | | ***** | |

**Supplementary information S1: Dietary contents**

**Altromin 1324 diet contents**


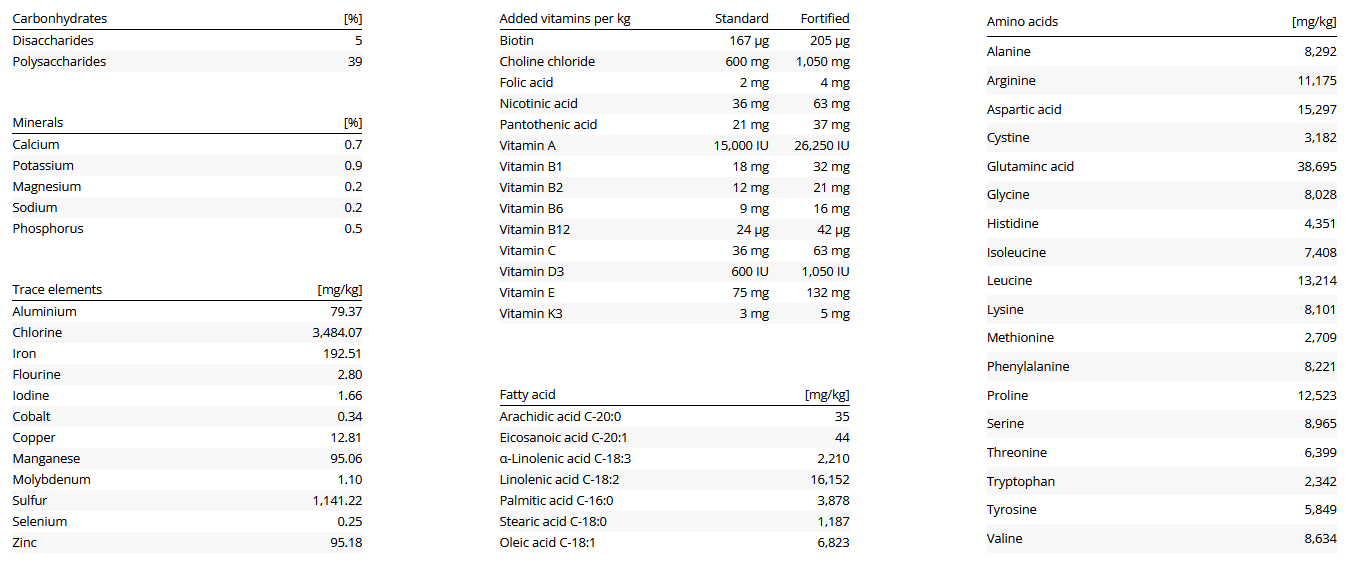


**High-fat D12451 diet contents**

| **Class description** | **Ingredients** | **Grams** |
| --- | --- | --- |
| Protein | Casein, Lactic, 30 Mesh | 200.00 g |
| Protein | Cystine, L | 3.00 g |
| Carbohydrate | Sucrose, Fine Granulated | 176.80 g |
| Carbohydrate | Lodex 10 | 100.00 g |
| Carbohydrate | Starch, Corn | 72.80 g |
| Fiber | Solka Floc, FCC200 | 50.00 g |
| Fat | Lard | 177.50 g |
| Fat | Soybean Oil, USP | 25.00 g |
| Mineral | S10026B | 50.00 g |
| Vitamin | Choline Bitartrate | 2.00 g |
| Vitamin | V10001C | 1.00 g |
| Dye | Dye, Red FD&C #40, Alum. Lake 35-42% | 0.05 g |
|  | Total: | 858.15 g |
